# Morphometric and volumetric analysis of the frontal sinus in a Brazilian population using Cone beam Computed Tomography: a forensic approach for sex, age and facial morphology estimation

**DOI:** 10.5281/zenodo.15564344

**Published:** 2025-08-01

**Authors:** Letícia Bego de Miranda, Beatriz Caio Felipe, Matheus Kawana Couto, Wilton Mitsunari Takeshita, Lilian Cristina Vessoni Iwaki, Mariliani Chicarelli da Silva

**Affiliations:** ^1^Department of Dentistry, State University of Maringá - Maringá, Parana, Brazil, ^2^Department of Oral Diagnosis, Piracicaba Dental School, State University of Campinas - Piracicaba, Sao Paulo, Brazil, ^3^Departament of Surgery and Diagnosis, Araçatuba Dental School, Sao Paulo State University, Araçatuba, Sao Paulo, Brazil.

**Keywords:** Computed tomography, Frontal sinus,, Forensic identification, Forensic dentistry

## Abstract

The aim of this study was to evaluate the linear and volumetric measurements of the frontal sinus (FS), using cone beam computed tomography (CBCT) scans, for a discriminatory analysis of gender, age and facial skeletal pattern in a Brazilian population. A total of 300 CBCT scans were analyzed, measuring the height, width, length and volume of the FS. The measurements were divided into groups: sex, age (20-24.9, 25-29.9, 30-34.9, 35-39.9, 40-45.9, 46-49.9, 50-54.9, 55-59.9, and 60-64.9 years) and facial skeletal pattern classes I, II and III. The function values in the centroid group were 0.675 for male and -0.292 for female. A rule was established indicating that if the value of D is greater than 0.19, the sample will be classified as male. The results showed a significant difference in women, who had significantly lower volume, width and depth than men (p<0.001, p=0.003, and p<0.001, respectively). No significant differences could be observed between the age and facial skeletal pattern groups. The results suggest that the FS measures of volume, height, width and depth have moderate discriminatory power for predicting gender in a Brazilian population. In conclusion, the results show that the FS has potential for assessing gender, but the accuracy of the method and its applicability for analyzing age and facial skeletal pattern were limited in our population.

## INTRODUCTION

Human identification plays a fundamental role humanitarianly and in procedural law, especially in civil and criminal cases. In the case of criminal investigations, natural disasters, plane crashes, cases of disappearances, (*2*, *3*) where it is not possible to apply conventional identification methods, the analysis of images of the anatomical structures of the skull becomes extremely important. (*4*) Because the skull, in addition to being considered the second best bone structure for age estimation, has the advantage of being highly resistant to damage. (*4*)

A particularly relevant anatomical structure in the cranial region is the frontal sinus (FS). It is characterized by a great variety and asymmetry and is unique to each individual. (*4*) Because it is protected by skull bones, it is resistant to trauma, presenting a high probability of remaining intact during catastrophes and mass accidents, thus allowing the identification of individuals and sex estimation. (*5*) In addition to sex estimation, assessing age and skeletal class are relevant aspects.

Globally, the literature is scarce in studies that explore the FS using cone beam computed tomography (CBCT) to estimate age, (*6*) sex or facial skeletal pattern. Although this topic has been little studied in the literature so far, only a limited number of studies addressing sex were found (*7*-*9*) and none of them investigated its relationship with forensic identification. Even though there are investigations in the area, to our knowledge, only two studies have evaluated the FS as an anatomical repair that can be used in human identification in a Brazilian population. (*10*, *11*)

The current literature regarding CBCT has reinforced the significant distinction in the morphological characteristics of FS in relation to sex. (*1*, *4*, *12*, *13*) However, there are studies that question this reliability of the FS with identification methods,14-17 many of which employ bidimensional (2D) scans and in some cases, resort to the use of dry skulls.

According to what has been studied in the literature up to date, there have been few volumetric analyses of the FS in relation to the age of adult individuals, which showed an expansion up to 40 years of age and a tendency to decrease with aging. (*18*, *19*) Regarding the relationship between the FS and facial skeletal pattern, there are divergences in the literature. While Sawada et al. (2022) found no significant association, these results contrast with previous studies. (*7*-*9*)

Therefore, the objective of this work was to evaluate the linear and volumetric measurements of the FS using CBCT, in order to establish a discriminative functional analysis for estimating sex, age and facial skeletal pattern in a Brazilian population. This study aims to fill a significant gap in the literature by providing valuable stimuli for the practical application of FS in forensic science by contributing to forensic databases.

## MATERIALS AND METHODS

This work was sent and approved by the Permanent Ethics Committee for Research Involving Human Beings at the State University of Maringá (UEM) under number CAAE: 68966523.2.0000.0104. Due to the retrospective nature of this study, signed informed consent was not required by the Committee.

### Sample selection

A total of 300 CBCT scans from anonymous Brazilian individuals were included in the research, divided into the following groups: sex, age (20-24.9, 25-29.9, 30-34.9, 35-39.9, 40-45.9, 46-49.9, 50-54.9, 55-59.9, and 60-64.9) and facial skeletal pattern (Class I (0 ° < ANB < 4°), Class II (ANB ≥ 4°) and Class III (ANB ≤ 0°) using Steiner classification. (*19*, *20*) The used scans belong to the archive of the Clinical Imaging Research Laboratory of the State University of Maringá (UEM) and were carried out as an indication for the most varied dental treatments.

The exclusion criteria were malformations, obliterations, pathologies and diseases, trauma, absence of the frontal sinus on one side and patients under 20 years of age. (*21*) As a result, 64 scans were excluded from the sample, therefore resulting in 235 scans to be analyzed. The final sample distribution is represented by tables 1 and 2.

**Table 1:** Sample division according to sex and age

**Group**	**Male**	**Female**	**Total**
20-24.9	23	37	60
25-29.9	11	39	50
30-34.9	7	13	20
35-39.9	7	19	26
40-45.9	6	13	19
46-49.9	6	19	25
50-54.9	5	11	16
55-59.9	4	6	10
60-64.9	3	6	9

**Table 2:** Sample division according to sex and facial skeletal pattern

**Sex**	**Facial Skeletal Pattern**		
**Class I**	**Class II**	**Class III**
Male	n=35	n=17	n=20
Female	n=54	n=68	n=43
Total	n=89	n=83	n=63

### Image acquisition

The scans were performed at the Clinical Imaging Research Laboratory (LIPC) of the Health Technology Center (CTS), of the Research Support Center Complex (COMCAP), located in the Department of Dentistry of the State University of Maringá (DOD/UEM) by the same oral and maxillofacial radiology team and are archived. They were obtained using the i-CAT Next Generation® equipment (Imaging Sciences International, Hatfield, PA, USA), with a volume of 300μ isometric voxel, FOV (Field of View) of 17 × 23 cm, tube voltage of 120 kVp and tube current of 3-8 mA. For the analysis, the Dolphin 3D Imaging software (Dolphin Imaging & Management Solutions®, Chatsworth, CA, USA) version 11.9 was used.

### Image analysis

For FS analyses, CBCT scans were exported using the Digital Imaging extension in Communications in Medicine (DICOM) and were imported into the Dolphin 3D Imaging software (Dolphin Imaging & Management Solutions®, Chatsworth, CA, USA), version 11.9. For each scan, the reconstructions were oriented, positioning the Frankfurt plane (line that passes horizontally from the upper edge of the external auditory meatus to the lower orbital edge) parallel to the horizontal plane in the sagittal reconstruction before measurements.

**Figure 1:**
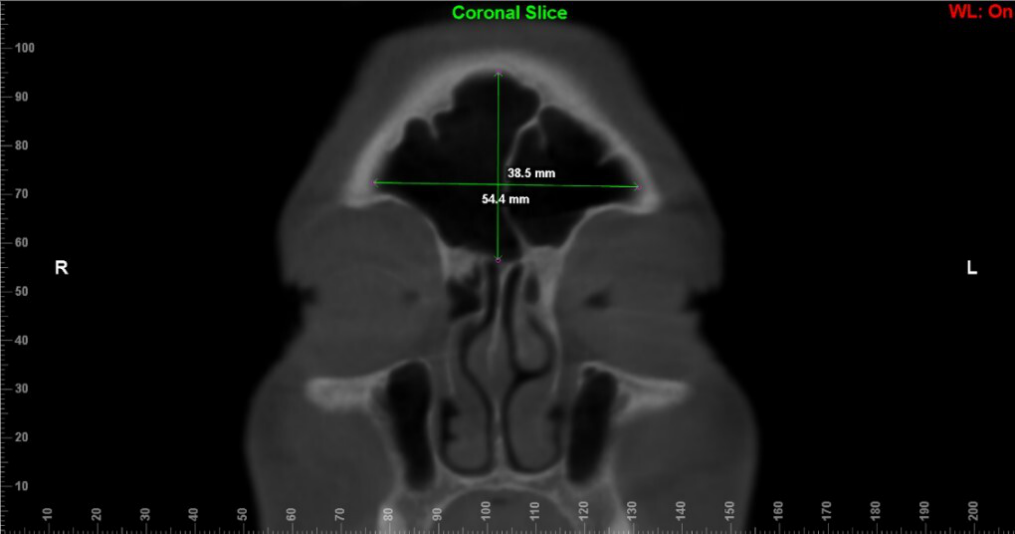
Linear dimensions for supero-inferior (vertical line) and medio-lateral (horizontal line) length of the FS

**Figure 2:**
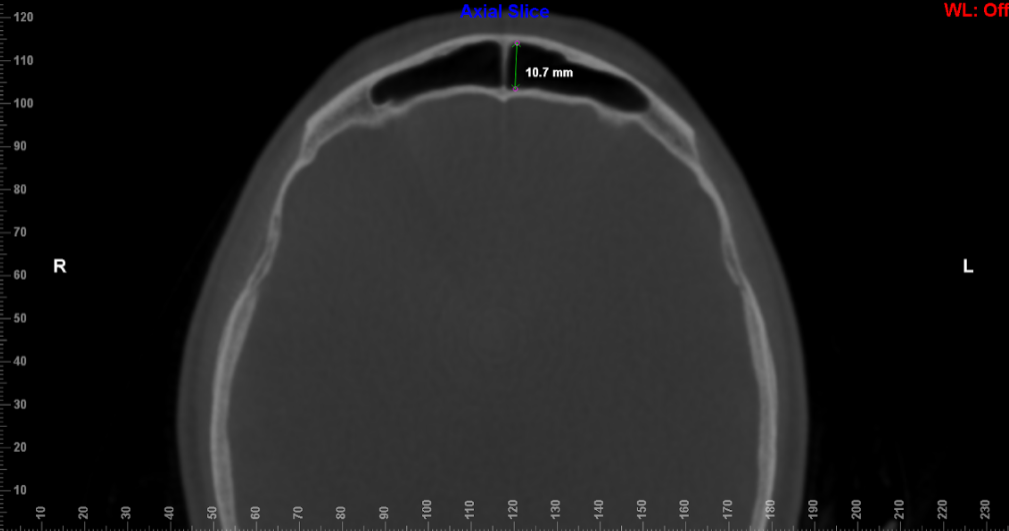
Linear dimension of the antero-posterior length of the FS

The evaluators underwent calibration through 10 CBCT scans. These assessments took place individually, randomly and in a dark and silent environment. Calibration lasted two weeks to ensure its reliability. The results were compared and discussed among the evaluators before the official measurements began. Linear and volumetric measurements were performed on 236 CBCT scans by two independent oral and maxillofacial radiologists (with more than 5 years of experience), blind to details of age, sex and facial skeletal class. To avoid eye fatigue, the two examiners evaluated only 10 scans per day.In order to assess sex, age and facial skeletal class, the following measurements were defined: height, width and length. These linear measurements (height, width and length) were taken in the coronal and axial reconstructions in the widest area of ​​the FS13, and the volume of the FS was acquired by delimiting its anatomy in the three reconstructions (axial, coronal, and sagittal), both using the Dolphin® software. (*22*, *23*) In the coronal reconstruction, the width was measured as the greatest distance between the medial and lateral walls of the FS, and the height as the greatest distance between the FS floor and ceiling (Figure 1). The depth was measured in the axial reconstruction as the distance from the anterior wall of the sinus to the posterior wall (Figure 2). (*13*)

**Figure 3:**
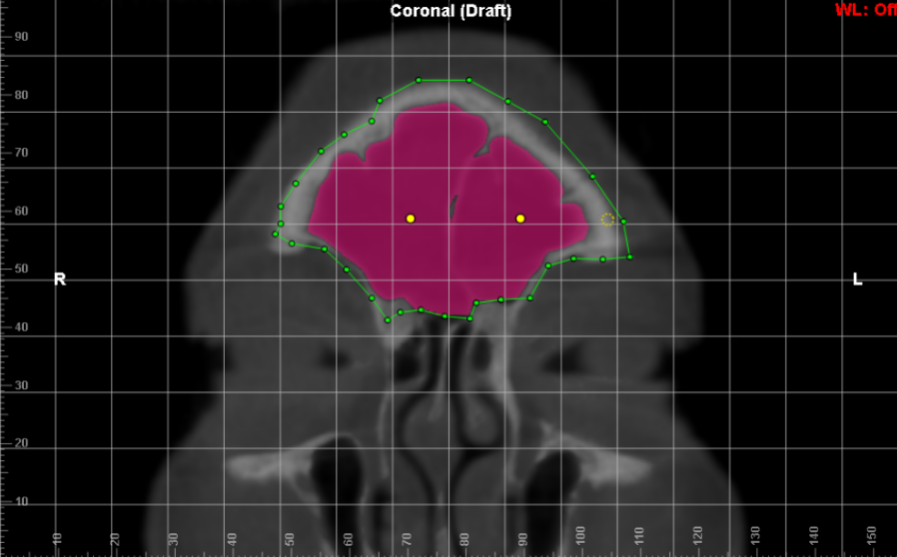
Delimitation of the anatomical borders of the FS in coronal reconstruction

**Figure 4:**
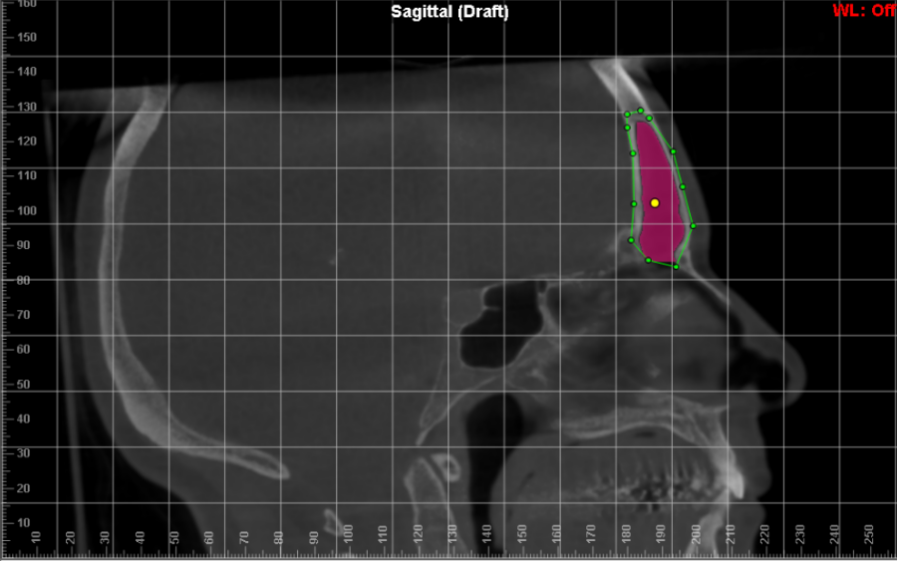
Delimitation of the anatomical borders of the FS in sagittal reconstruction

**Figure 5:**
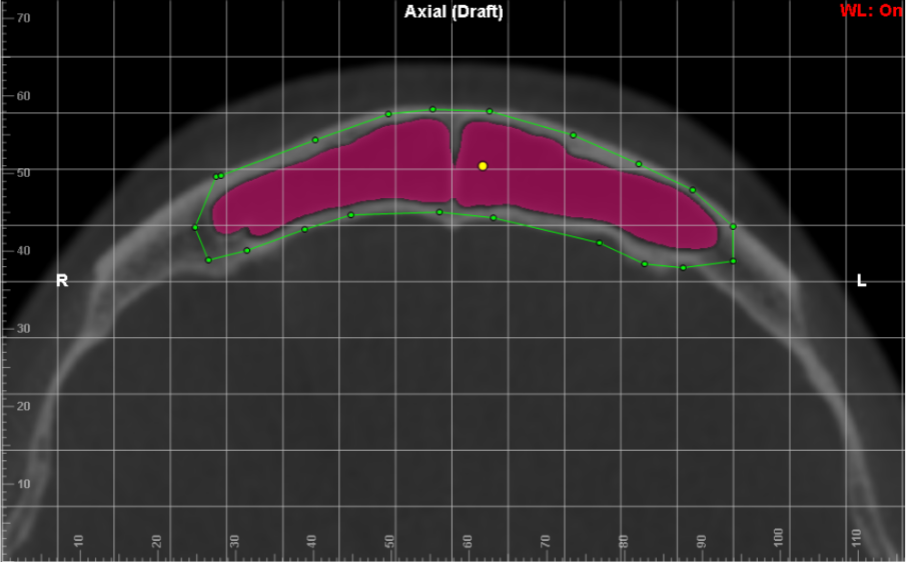
Delimitation of the anatomical borders of the frontal sinus in axial reconstruction

To analyze the FS volume, the sinus/airway tool of the Dolphin® software was used as it allows reliable measurements, which produces a complete filling of the delimited region, preventing differences in area from interfering with the reliability of the data. (*23*) In order to correctly segment the entire FS, regardless of its anatomical variability, the first step was to delimit the region of interest with points (green dots) that consequently formed a line, on its anatomical border in the coronal reconstruction (Figure 3). Afterwards, the exact delimitations in the other planes were checked (Figures 4 and 5), if any point was outside the delimitation, it could be changed. (*23*) After this, “seed points” (yellow dots) were inserted in the frontal sinus, whose function is to expand within the airway, up to the selected limit, and when necessary, more “seed points” can be added.

Before the final volumetric calculation, the sensitivity threshold was also adjusted in all image acquisitions, being a tool that controls the filling of the volume of the region of interest. (*24*) Although the sensitivity threshold is automatically determined by the Dolphin® software, (*25*) for the present study the average value was ±50, adapted for the FS.

**Figure 6:**
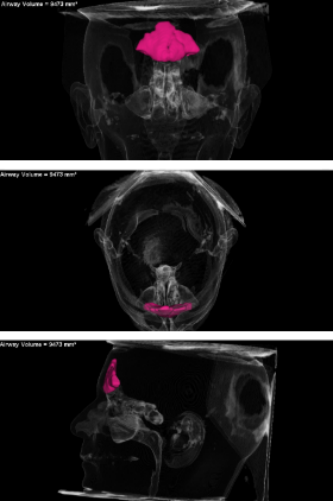
3D model generated by the Dolphin 3D Imaging software

After complete segmentation of the region of interest, the Dolphin® software automatically displayed the volume in cubic millimeters (mm^3^) of the selected area and a three-dimensional (3D) model of the FS (Figure 6). All viewing plans were checked to ensure that the enclosed area had been completely filled. (*26*, *27*) All images were analyzed following the established sequence protocol: linear measurements, volumetric analysis and sex, age and facial skeletal class comparison with the patient’s known data.

### Statistical analysis

A database for qualitative and quantitative variables was organized to allow tabulation and statistical analysis. All statistical procedures were calculated using the statistical programs SPSS 25.0 (SPSS Inc., Chicago, IL, USA) and Bioestat 5.3 (Instituto Mamirauá, Pará, Brazil). Initially, a descriptive statistical analysis was carried out to obtain absolute and relative numbers.

The Intraclass Correlation Coefficient (ICC) was used to evaluate intra- and inter-observer agreement. To evaluate the Gaussian distribution of the data, the Shapiro-Wilk test was performed and the Mann-Whitney test was applied for independent variables, between sexes, for measurements of the FS region. Regarding the facial skeletal pattern, the analysis of variance (ANOVA) was applied to compare the different FS analyses. For the age, Kruskal-Wallis was applied to compare the different FS analyses. To develop a formula capable of estimating sex through FS measurements, a multivariate discriminant analysis was carried out using the direct method, and for this purpose, the Wilks’ lambda and Box’s M tests were applied.

## RESULTS

In table 3, it is possible to observe that the volume, width and depth of the FS showed significant differences between female and male. Female individuals exhibited significantly smaller volume, width and depth than men (p-value<0.001, 0.003 and <0.001, respectively).

**Table 3:** FS parameters according to sex

	**Sex**	**N**	**Mean**	**Median**	**SD**	**p-value**
Volume (mm^3^)	F	164	4579.76	3966.00	2739.13	<0.001*
	M	71	7288.35	6344.00	4366.16	
Height (mm)	F	164	24.03	25.05	8.49	0.958
	M	71	24.26	25.50	7.74	
Width (mm)	F	164	38.04	40.55	11.55	0.003*
	M	71	42.87	45.70	13.39	
Depth (mm)	F	164	7.34	7.30	2.19	<0.001*
	M	71	9.37	9.10	2.49	

Mann-Whitney test; SD (standard deviation); *p<0.05

In tables 4 and 5, no statistically significant differences were found between the groups separated by age and facial skeletal pattern.

**Table 4:** FS parameters according to sex

	**Group**	**N**	**Mean**	**Median**	**SD**	**p-value**
Volume	20-24.9	60	5662.03	5335	475.684	0.240
	25-29.9	50	5419.88	4262	503.215	
	30-34.9	20	6459.90	5509	939.075	
	35-39.9	26	6058.31	4302	842.146	
	40-45.9	19	5405.37	6070	690.354	
	46-49.9	25	4165.20	3769	543.905	
	50-54.9	16	3715.06	2823	783.026	
	55-59.9	10	5064.11	5056	785.282	
	60-64.9	9	5601.33	5433	712.646	
Height	20-24.9	60	24.53	26.8	1.043	0.127
	25-29.9	50	23.20	24.5	1.159	
	30-34.9	20	25.93	25.9	1.843	
	35-39.9	26	25.97	26.2	1.502	
	40-45.9	19	25.95	26.4	2.157	
	46-49.9	25	21.83	23.7	1.796	
	50-54.9	16	19.49	19.0	2.334	
	55-59.9	10	23.52	23.8	1.718	
	60-64.9	9	27.04	26.3	1.288	
Width	20-24.9	60	39.62	40.5	1.600	0.119
	25-29.9	50	39.61	41.1	1.581	
	30-34.9	20	43.79	42.5	2.516	
	35-39.9	26	40.57	41.3	2.365	
	40-45.9	19	40.18	43.3	3.288	
	46-49.9	25	35.61	40.9	2.668	
	50-54.9	16	32.61	34.0	3.536	
	55-59.9	10	40.48	43.3	3.711	
	60-64.9	9	44.20	43.4	1.843	
Depth	20-24.9	60	8.15	8.40	0.333	0.983
	25-29.9	50	8.00	8.15	0.386	
	30-34.9	20	7.99	8.40	0.482	
	35-39.9	26	8.07	7.45	0.491	
	40-45.9	19	7.51	8.30	0.547	
	46-49.9	25	7.80	7.80	0.498	
	50-54.9	16	7.53	8.25	0.632	
	55-59.9	10	8.12	8.80	0.695	
	60-64.9	9	7.67	7.20	0.480	

Kruskal-Wallis test; SD (standard deviation); *p<0.05

**Table 5:** FS parameters according to facial skeletal pattern

	**Class**	**N**	**Mean**	**SD**	**p-value**
Volume (mm^3^)	I	89	5289.67	3176.82	0.932
	II	83	5443.00	3567.69	
	III	63	5492.13	3990.49	
Height (mm)	I	89	23.94	7.56	0.073
	II	83	25.54	8.70	
	III	63	22.40	8.38	
Width (mm)	I	89	39.70	12.06	0.980
	II	83	39.35	12.13	
	III	63	39.40	13.06	
Depth (mm)	I	89	7.91	2.50	0.084
	II	83	8.38	2.38	
	III	63	7.47	2.46	

ANOVA test; SD (standard deviation); *p<0.05

Table 6 shows the Wilks' Lambda test, applied to evaluate the ability of the discriminant equation to distinguish the sexes based on the values ​​of the FS volume, height, width and depth analyses. The Wilks' Lambda value was 0.834, with a p-value of <0.001, indicating that the discriminant equation is statistically significant and has discriminatory power to distinguish the sexes. Furthermore, it is possible to observe that the overall percentage of precision in predicting gender was 74.5%, which indicates a moderate capacity for accuracy in identifying gender based on the measurements considered.

**Table 6:** Discriminant analysis of sex, using the measures in discrimination

**Total (sex)**			
D = -1.125 + 0.00*volume -0.005*height - 0.001*width + 0.12*depth			
Wilks’ Lambda = 0.834, p<0.001			
Percentage of accuracy in predicting sex			Overall
			74.5%
Functions at centroid group	Male	Female	Male
	0.675	-0.292	if D > 0.19

The function values in the centroid group were 0.675 for males and -0.292 for females. A rule was established indicating that if the D value is greater than 0.19, the sample will be classified as male. These results suggest that FS volume, height, width and depth measurements have moderate discriminatory power to predict sex in a Brazilian population.

## DISCUSSION

To our knowledge, this is the first study to discuss morphometric measurements of FS in relation to age, sex and skeletal class in a Brazilian population. We observed that the FS showed significant differences between the sexes, with females exhibiting smaller volume, width and depth compared to males (p-value<0.001, 0.003 and <0.001, respectively). However, it was not possible to observe statistically significant differences in relation to age and skeletal class.

Although some studies have explored the volume of FS in assessing sex, its practical application in forensic anthropology is still in its infancy. (*1*) In the current scientific literature, based on helical computed tomography (CT) or CBCT, the significant distinction in the morphological characteristics of SF in relation to sex is reinforced. (*1*, *4*, *12*, *13*, *28*, *29*) Nevertheless, there are works that question the reliability of FS as a forensic identification method, (*14*-*17*) many of which employ 2D examinations and in some cases, resort to the use of dry skulls. In this present approach, using CBCT, the limitations of two-dimensional images can be surpassed, considering that 3D images provide details without superimposition, improving analysis and measurements, allowing more accurate results. (*11*)

Regarding age, our results did not indicate statistically significant correlations, suggesting a complex relationship between FS and aging. Even so, there are some studies that have shown growth of the FS until the age of 40, and its morphology can be influenced due to factors such as mechanical stress and growth hormones. (*18*, *19*) Our sample, with n=9 for the group over 60 years old, did not reflect this correlation. This points to the need for more comprehensive investigations involving different age groups.

Exploring the correlation between FS and facial skeletal pattern, the results of this research did not reveal statistically significant associations, similar to the pilot study by Sawada et al. (*9*) However, it differed from two previous studies. (*7*, *8*) Metin-Gürsoy et al. (2021) when performing linear measurements on CBCT with individuals aged 17 to 38 years, found that the anteroposterior dimension and width were significantly smaller in the hyperdivergent group compared to the hypodivergent, revealing a correlation between FS and craniofacial measurements. Rossouw et al. (1991) analyzed the FS using 2D cephalometry and concluded that there is a significant relationship between this structure and skeletal class. Therefore, our results suggest that FS may not be a reliable indicator for facial skeletal pattern, highlighting the complexity of these relationships and the need for additional investigations. It is worth highlighting that variations in the size and capacity of the FS may be related to its development during puberty, in parallel with craniofacial growth. (*7*)

The present study has some limitations, such as the convenience sample and individuals aged at least 20 years. (*1*) This age is considered the end of the FS growth. For this reason, measurements and comparisons were not considered in people of younger age. (*1*) Expanding the sample is necessary to better represent a population. (*1*) The present sample showed an inequality between the facial skeletal pattern and age groups, therefore requiring the expansion of the sample, following standardized protocols for different populations. However, the search for a reliable, accessible and accurate method for human identification motivates the continuation of these studies, providing valuable information for forensic science.

This analysis provided an accuracy of 74.5% in sex estimation using a single anatomical structure. This is a moderate index, but it can be used to estimate an individual's sex when other identification methods cannot be used. Furthermore, this expanded study not only corroborates the previous conclusions (*30*), but also deepens the discussion on the challenges and advances in FS forensic analysis. The application of CBCT and the volumetric assessment of FS show promise, but there is still the need for more comprehensive and diversified research to consolidate its role in forensic science, especially in populations as mixed as Brazil’s.

## CONCLUSION

This analysis provided a moderate index of 74.5% in sex estimation using a single anatomical structure. It shows that it is possible to use the FS in forensic analysis for sex estimation when there are no other identification methods available. No statistically significant correlations were found between the FS and age or facial skeletal pattern. Future investigations with a broader age interval and more ethnically varied samples are recommended to improve the understanding of these relationships and consolidate the role of FS in forensic science.
